# Dual Drug-Loaded Enzyme-Responsive Liposomes Exert Specific Modulation on Liver Cancer Cells and Tumor-Associated Macrophages In Vitro

**DOI:** 10.3390/pharmaceutics18080964

**Published:** 2026-08-05

**Authors:** Shudong Zhang, Jun Yan, Shiyu Zhu, Xinchen Liu, Lele Wang, Yuhang Liu, Yan Wei

**Affiliations:** 1 Institute of Translational Medicine, Shanghai University, Shanghai 200444, China; 2 MedEng-X Institutes, Shanghai University, Shanghai 200444, China

**Keywords:** hepatocellular carcinoma, in situ vaccination, TAMs, sPLA_2_-responsive liposomes, remote loading methods

## Abstract

**Background**: Hepatocellular carcinoma (HCC) is a highly lethal malignancy and remains a major contributor to global cancer mortality. In situ vaccination (ISV) holds great potential for HCC therapy. Still, its efficacy is severely limited by intrinsic antigen scarcity and the tumor-associated macrophage (TAM)-mediated inhibition of dendritic cell (DC) maturation and T cell activation. In particular, the M2 TAM-HCC cell crosstalk may further aggravate ISV suppression. Moreover, as an internal organ tumor, HCC requires systemic administration of ISV agents, which often cause severe off-target toxicity. **Methods**: To address these challenges, we developed secretory phospholipase A2 (sPLA_2_)-responsive liposomes co-loaded with the TLR7/8 agonist R848 and the ICD inducer doxorubicin (DOX) via a sequential remote loading method, termed sP-Lips@DR. **Results**: Incorporation of cholesterol (CHOL) significantly improved DOX encapsulation, and the sequential loading method enabled efficient co-encapsulation of both R848 and DOX. sP-Lips@DR responds to an sPLA_2_-overexpressed, mildly acidic microenvironment in HCC, enabling selective drug release. In addition, sP-Lips@DR exhibited sPLA_2_-responsive cytotoxicity toward H22 cells and macrophage phenotypic shift. Furthermore, sP-Lips@DR could disrupt the crosstalk between M2-like macrophages and H22 cells in an sPLA_2_-responsive manner. **Conclusions**: Overall, the preparation and in vitro evaluation of sP-Lips@DR demonstrate their potential to enhance ISV efficacy in HCC, providing a solid foundation for future in vivo investigations.

## 1. Introduction

Hepatocellular carcinoma (HCC) is the major form of primary liver cancer, representing approximately 90% of diagnosed cases [1,2,3]. Although it ranks sixth in global incidence, HCC has the third-highest mortality rate due to its insidious progression and late diagnosis [4,5]. Inspired by the success of vaccines against infectious diseases, tumor vaccines have emerged as a novel cancer immunotherapy. Compared with traditional tumor vaccines, which are limited by challenges such as complex antigen identification and tumor heterogeneity [6,7], in situ vaccination (ISV) exhibits high specificity and efficacy. ISV induces immunogenic cell death (ICD), releasing a broad repertoire of endogenous tumor antigens for uptake and presentation by dendritic cells (DCs), thereby activating tumor-specific T cells [8,9]. Nevertheless, antigen scarcity and M2 TAM–mediated suppression of DC maturation and T-cell activation limit ISV efficacy in HCC. Specifically, tumor heterogeneity and immune selection pressure often drive antigen downregulation or loss [10,11]. TAMs are the most abundant immune cells in HCC (~17.7% of infiltrating immune cells) and predominantly exhibit a pro-tumor M2 phenotype [12,13,14]. M2 TAMs suppress DC maturation via IL-10/JAK/STAT3 signaling [15] and impair T-cell function through the upregulation of PD-L1 and CTLA-4 [16,17], arginase secretion [18], and TREM-1 upregulation [19]. Moreover, crosstalk between M2 TAMs and HCC cells may reinforce M2 polarization and further impair ISV efficacy [13]. Thus, to realize the full potential of ISV, it is essential to overcome these limitations in HCC.

TLR 7/8 agonist, R848, repolarizes M2 TAMs toward the M1 phenotype [20], restoring DC maturation and T cell activation. DOX induces ICD, releasing tumor antigens and danger-associated molecular patterns (DAMPs) that promote DC activation and antigen presentation [21,22]. Hence, we speculated that the combination of R848 and DOX may achieve potent ISV efficacy in HCC. Unfortunately, systemic administration of R848 can trigger immune-related toxicity [23], while DOX exhibits dose-limiting toxicity in normal tissues [24,25]. To avoid systemic toxicity, all currently approved ISVs are locally administered to superficial or accessible tumors [26,27]. In contrast, HCC, as a deep-seated tumor, requires systemic administration. Therefore, a nanoplatform with preferential tumor accumulation and tumor-specific drug release is needed for ISV while minimizing side effects to normal tissues.

Liposomes, with excellent biocompatibility, the ability to reduce systemic toxicity, and controlled-release properties, are considered ideal drug delivery vehicles [28]. In particular, enzyme-responsive liposomes can respond to the overexpressed enzymes in the TME for site-specific drug release [29,30]. Secreted phospholipase A2 (sPLA_2_) is highly expressed in various tumors [31,32], with levels in HCC up to 5-fold higher than in normal tissues [33]. By hydrolyzing the ester bond at the sn-2 position of lipids, such as DPPC, sPLA_2_ disrupts lipid structures [34], making it an effective endogenous stimulus for drug release. In addition, DOX and R848 belong to weak bases [35], and remote loading is expected to enable efficient encapsulation and stable retention of weakly basic drugs. For example, remotely loaded DOX forms gel-like precipitates with intraliposomal sulfate ions, thereby minimizing drug leakage [36,37]. As such, sPLA_2_-responsive liposomes with remotely loaded drugs would achieve stable drug encapsulation and enzyme-triggered, tumor-specific drug liberation, representing a promising nanoplatform for ISV. However, few studies have reported an enzyme-responsive liposome remotely loaded with dual drugs.

**Scheme 1:**
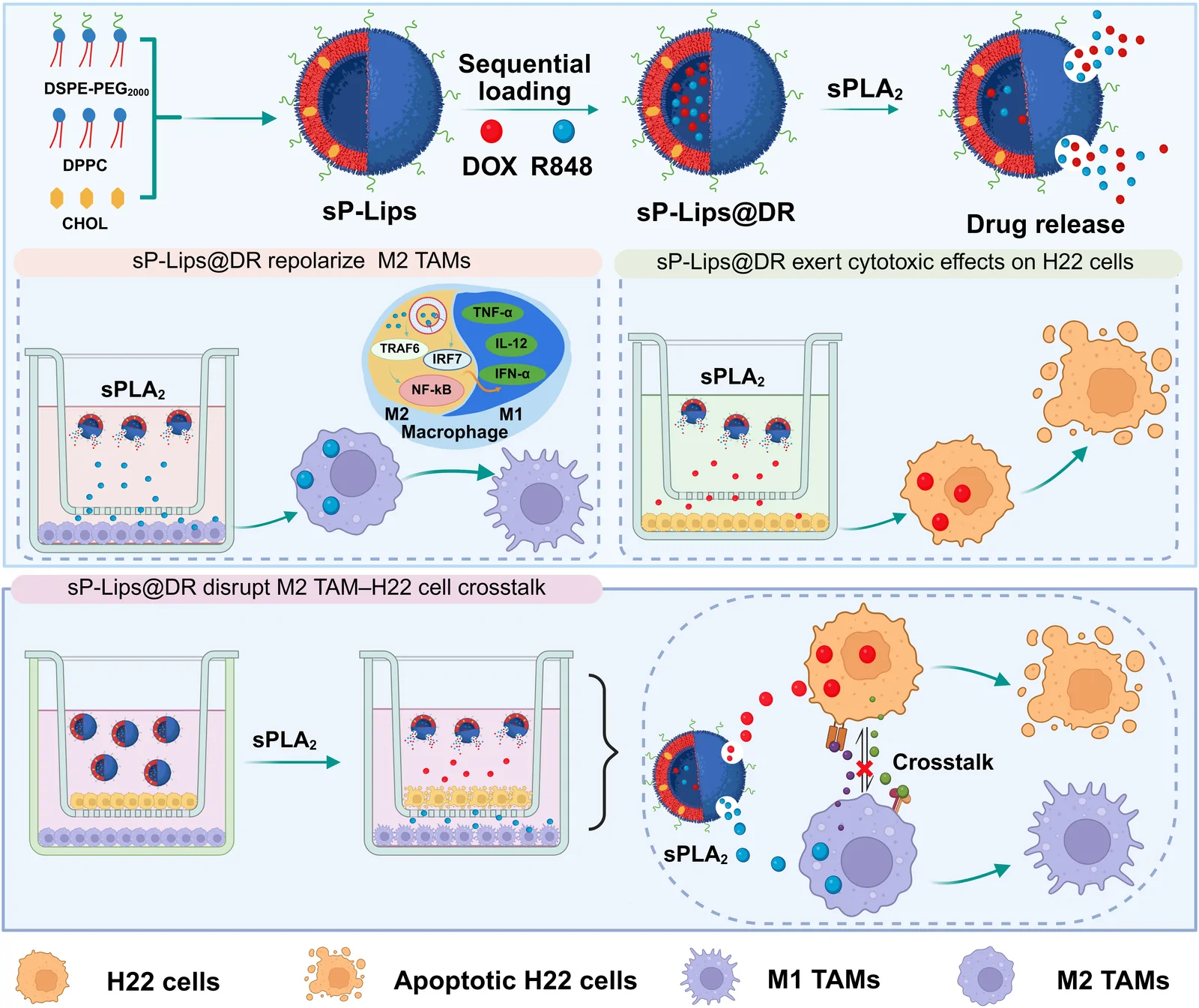
Schematic illustration of the preparation and mechanism of sP-Lips@DR for ISV treatment of HCC. sP-Lips@DR selectively release dual drugs in response to sPLA_2_, effectively repolarizing M2 macrophages and promoting H22 cell apoptosis. Furthermore, sP-Lips@DR can disrupt the M2 TAMs-H22 cell crosstalk in an sPLA_2_-dependent manner.

In this study, we designed sPLA_2_-responsive, dual-drug-loaded liposomes, sP-Lip@DR (Scheme 1). Formulation screening data showed that CHOL significantly enhanced drug encapsulation efficiency (EE), and a sequential loading process achieved higher EE for each drug than a co-loading process. sP-Lips@DR enable sPLA_2_-dependent cellular uptake, selectively modulating M2 macrophages and specifically killing H22 cells. Furthermore, sP-Lips@DR can disrupt the crosstalk between M2 TAMs and H22 cells in an sPLA_2_-dependent manner. The properties of enzyme-promoted dual-drug release and efficacy make the liposome a promising nanoplatform for ISV.

## 2. Methods

### 2.1. Preparation and Characterization of sP-Lips@DR

#### 2.1.1. Preparation of sP-Lips@DR

The liposomes were prepared using the thin-film hydration method. The lipid components were dissolved in chloroform, followed by solvent removal under vacuum through rotary evaporation. The thin lipid film was subsequently hydrated with 800 μL of ammonium sulfate solution (200 mM). The liposome suspension was subjected to pulsed ultrasonication at 200 W for 1 min, using an on/off cycle of 1 s/2 s. To create a pH gradient, the liposome suspension was passed through a Sephadex CL-4B column, yielding liposomes with ammonium sulfate solution as the internal aqueous phase and PBS (pH 7.4) as the external aqueous phase. Drug loading was performed using a remote-loading method at 39 °C. DOX or R848 was added to 1 mL of the liposome suspension and incubated for 40 min. Unencapsulated DOX or R848 was removed by ultracentrifugation (200,000× *g*, 2 h). The liposomal pellet was resuspended in PBS to obtain the drug-loaded liposomes.

#### 2.1.2. Drug Loading Capacity and EE

The liposomes were mixed with methanol at a volume ratio of 1:3, followed by 10 min of sonication. After centrifugation at 14,000 rpm for 10 min, the supernatants were collected for fluorescence intensity determinations of DOX or R848 using a microplate reader (Infinite^®^ 200 PRO, Tecan, Männedorf, Switzerland). Drug concentrations were calculated from the standard curves. Drug loading capacity (DLC) and EE were calculated based on the following equations.

(1) $$DLC\left(\%\right)\;=\;\frac{amount\;of\;DOX/R848\;encapsulated\;within\;the\;liposomes}{amount\;of\;DOX/R848\;and\;liposomes}\;\times \;100$$

(2) $$EE(\%)\;=\;\frac{amount\;of\;DOX/R848\;encapsulated\;within\;the\;liposomes}{amount\;of\;fed\;DOX/R848}\;\times \;100$$

#### 2.1.3. Screening of Liposomal Formulations

DPPC and DSPE-PEG_2000_ (95:5, mol/mol) were used for sP-Lips formulation 1 (termed sP-Lips (F1)), and DPPC, CHOL, and DSPE-PEG_2000_ (75:20:5, mol/mol) were used for sP-Lips formulation 2 (termed sP-Lips (F2)).

As described above, liposomes were fabricated via thin-film hydration by dissolving the lipid components in chloroform. To prepare DOX-loaded liposomes, 0.5 mg of DOX was introduced into the liposome suspension for 40 min of incubation. Unencapsulated drugs were removed through ultracentrifugation. The formulations were screened based on their EE and DLC. Finally, sP-Lips (F2) were selected for subsequent use due to superior drug encapsulation efficiency.

#### 2.1.4. Optimization of Preparation Processes for Dual-Drug-Loaded Liposomes

The preparation process of sP-Lips@DR was further optimized. For simultaneous loading, R848 (0.1 mg) and DOX (0.5 mg) were simultaneously added to 1 mL of the sP-Lips suspension for 40 min of incubation. For sequential loading, R848 (0.1 mg) and DOX (0.5 mg) were sequentially added to the liposome suspension at a time interval of 40 min, allowing each step 40 min of incubation. The unencapsulated drug was removed by ultracentrifugation (200,000× *g*, 2 h). The optimal preparation process was screened based on the EE and DLC.

#### 2.1.5. Characterization of sP-Lips@DR

The optimized formulation of sP-Lips@DR was characterized. The particle size and zeta potential of the liposomes were measured using a Zetasizer Advance Pro analyzer (Malvern, Worcestershire, UK). For particle morphological analysis, 5 μL of sP-Lips@DR suspension was dropped onto a 300-mesh carbon-coated copper grid and maintained for 5 min. This process was repeated three times. The particle morphology of sP-Lips@DR was visualized using a transmission electron microscope (TEM) (JEM-1400 Flash, JEOL, Tokyo, Japan).

### 2.2. Evaluation of the Drug Release Behavior of sP-Lips@DR

For the DOX release assay, free DOX or sP-Lips@DR were sealed in dialysis bags (MWCO = 3000 Da), immersed in the release media, and gently shaken at 37 °C. The release of free DOX was evaluated in PBS (pH 6.7), and sP-Lips@DR was assessed in 10% FBS (sP-Lips@DR (−E)) or in PBS (pH 6.7) containing sPLA_2_ (sP-Lips@DR (+E)). The final concentration of sPLA_2_ was 4.37 U/mL [33], and the same concentration was used in all subsequent experiments. At specified time points, a 200-μL aliquot was taken from the release medium, followed by supplementation with an equivalent volume of fresh medium. The fluorescence intensities of DOX (Ex/Em: 485/590 nm) in the collected samples were determined using a microplate reader (Infinite^®^ 200 PRO, Tecan, Männedorf, Switzerland). The DOX concentration was calculated through the calibration curves to profile the drug release curve.

R848 release from the liposomes was determined as described above. The fluorescence intensity of R848 in each sample was detected at λ_Ex/Em_ = 260/360 nm. The cumulative release of R848 was profiled accordingly.

### 2.3. Validation of sPLA2-Dependent Disruption Effects of sP-Lips@DR on M2 TAM–H22 Cell Crosstalk

#### 2.3.1. Validation of the Disruption Effects of DOX/R848 on M2 TAM-H22 Cell Crosstalk

H22 cells and M2 macrophages were seeded in the upper and lower chambers of a Transwell system, respectively, and incubated at 37 °C overnight. PBS, DOX, R848, or DOX + R848 was added to the upper chamber, followed by 48 h of incubation. H22 cells were harvested, and PI staining was performed at a concentration of 10 μg/mL for 30 min. The stained cells were washed with PBS and analyzed by flow cytometry assay (Cytoflex LX, Beckman, Indianapolis, IN, USA). In addition, PI-stained H22 cells were dispersed in 100 μL PBS and then loaded into a black 96-well plate. The fluorescence signals were detected using an in vivo spectrum imaging system (AniView100, BLT, Guangzhou, China) at the specified wavelengths (λ_Ex/Em_ = 465/600 nm).

M2 TAMs in the lower chambers were harvested and labeled with APC-conjugated anti-mouse CD206 and PE-conjugated anti-mouse CD86 antibodies and detected using a flow cytometry assay (Cytoflex LX, Beckman, Indianapolis, IN, USA). In addition, the cells were stained with FITC-conjugated phalloidin for 30 min, followed by DAPI staining for 10 min. Cell morphology was then visualized using CLSM (FV3000, Olympus, Tokyo, Japan).

#### 2.3.2. sPLA_2_-Responsive Modulation Effects of sP-Lips@DR on the Intercellular Crosstalk

The Transwell system was established as described above. PBS, sP-Lips@DR, or sP-Lips@DR/E was introduced into the upper chamber, followed by 48 h of culture. Afterward, H22 cells were stained with PI, followed by analysis using flow cytometry and an in vivo spectrum imaging system as described above. Meanwhile, M2 TAMs from the lower chambers were stained with APC-conjugated anti-mouse CD206 and PE-conjugated anti-mouse CD86 antibodies for flow cytometry analysis. In addition, the cells were stained with FITC-conjugated phalloidin and imaged using CLSM.

### 2.4. Statistical Analysis

Data are shown as mean ± SD. Statistical analyses were performed using GraphPad Prism 8.4.3. Significant differences between three or more independent groups were evaluated using a nonparametric two-tailed ANOVA, followed by Tukey’s post hoc test. Statistical significance was defined as * *p* < 0.05, ** *p* < 0.01, and *** *p* < 0.001.

## 3. Results and Discussion

### 3.1. Screening of Liposomal Formulations

**Figure 1:**
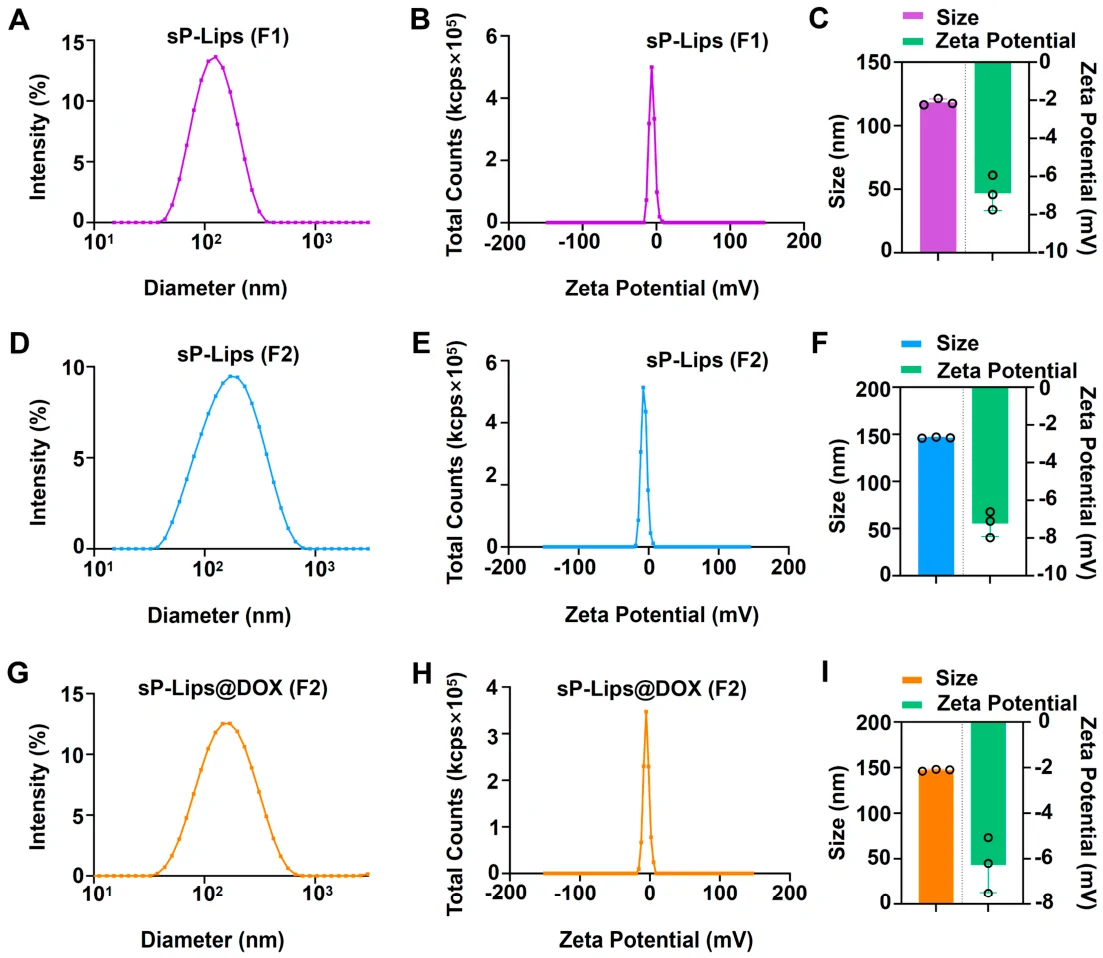
Screening of Liposomal Formulations of sP-Lips. (**A**) Particle size distribution and (**B**) zeta potential of sP-Lips (F1). (**C**) Quantitative analysis of particle size and zeta potential of sP-Lips (F1). (**D**) Particle size distribution and (**E**) zeta potential of sP-Lips (F2). (**F**) Quantitative analysis of particle size and zeta potential of sP-Lips (F2). (**G**) Particle size distribution and (**H**) zeta potential of sP-Lips@DOX (F2). (**I**) Quantitative analysis of particle size and zeta potential of sP-Lips@DOX (F2). Data were represented as mean ± SD (*n* = 3).

To achieve co-encapsulation of DOX and R848, we first screened the liposomal composition. PEGylation is commonly used to improve the circulation stability of liposomes. Herein, DSPE-PEG_2000_ was incorporated into our formulation at 5 mol%, consistent with that used in the marketed doxorubicin liposomal formulation Doxil^®^ [38]. sP-Lips (F1) were initially prepared using DPPC and DSPE-PEG_2000_ at a molar ratio of 95:5 (Table 1). As shown in Figure 1A–C and Table S1, sP-Lips (F1) exhibited an average particle size of 118.5 nm, a polydispersity index (PDI) of 0.226, and a negative surface charge (−6.87 mV), indicating good dispersibility and a suitable size for drug delivery.

Next, we prepared sP-Lips@D (F1) using the ammonium sulfate gradient method. To remove unencapsulated drugs, we first screened the appropriate ultracentrifugation speed [39,40]. As shown in Figure S1 and Table S3, when the centrifugation force was increased from 150,000× *g* to 200,000× *g*, the supernatant of sP-Lips@DiD (F1) displayed a lighter blue color with a recovery rate of 94.02%. These results indicated that the majority of liposomes could be collected under this force. Therefore, 200,000× *g* for 2 h was chosen for subsequent experiments. As shown in Table S4, the EE of DOX was only 15.08%, which was significantly lower than that of Doxil/Caelyx (≥95%), the first FDA-approved liposomal formulation of DOX [37]. This result indicated that DOX could not be efficiently encapsulated into sP-Lips (F1) via remote loading, possibly due to the relatively low phase transition temperature of DPPC (Tm ≈ 41 °C), which increases membrane fluidity [41] and consequently facilitates drug leakage.

Cholesterol (CHOL) can interact with phospholipid acyl chains and enhance lipid packing and acyl-chain ordering within the liposomal membrane, thereby reducing membrane fluidity [42], which is crucial for efficient drug encapsulation. Thus, we introduced CHOL into sP-Lips at 20 mol% to obtain sP-Lips (F2). As shown in Figure 1D,F, sP-Lips (F2) exhibited a particle size of 146.4 nm with a PDI of 0.245. In addition, we confirmed that a centrifugation force of 200,000× *g* was also suitable for the recovery of sP-Lips (F2) (Figure S2 and Table S3). The EE of DOX in sP-Lips@DOX (F2) was 94.35% (Table S4), demonstrating that the incorporation of CHOL significantly enhanced drug encapsulation efficiency. Moreover, the average particle size of sP-Lips@DOX (F2) was 147.2 nm, with a PDI of 0.252 and a zeta potential of −6.27 mV, indicating a desirable particle property (Figure 1G–I).

### 3.2. Optimization of Preparation Processes for Dual-Drug-Loaded Liposomes

**Figure 2:**
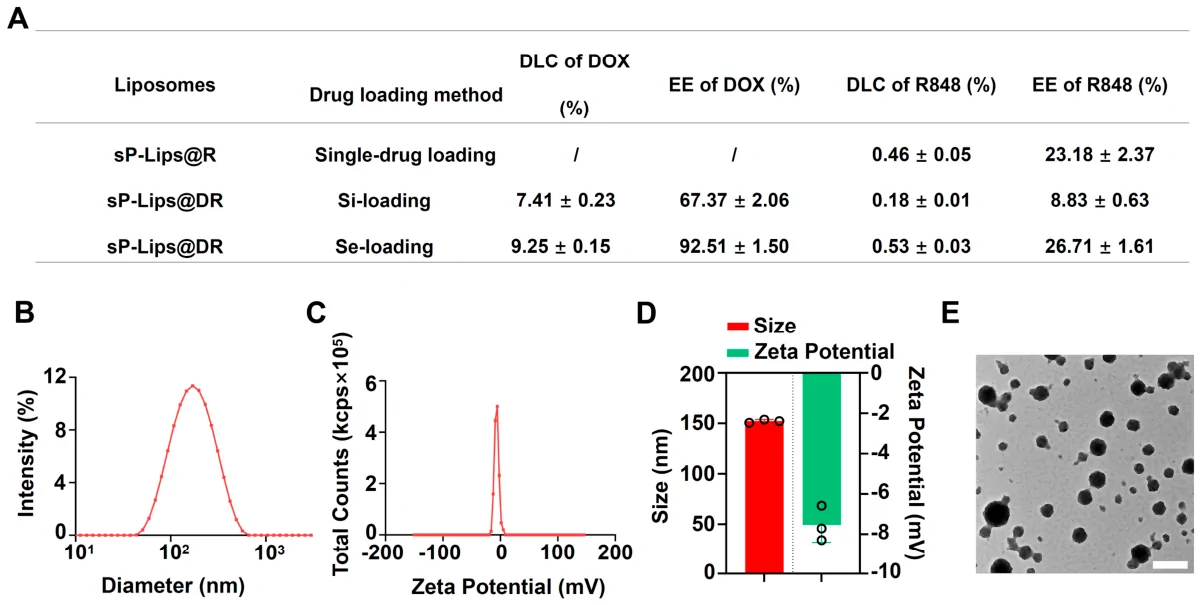
Preparation process, screening, and characterization of dual-drug-loaded liposomes. (**A**) Comparison of encapsulation efficiency (EE) and drug loading efficiency (DLE) of DOX or R848 in sP-Lips@DR under simultaneous loading (Si-loading) versus sequential loading (Se-loading) conditions. (**B**,**C**) Particle size distribution (**B**) and zeta potential (**C**) of sP-Lips@DR. (**D**) Quantitative analysis of particle size and zeta potential of sP-Lips@DR. Data were presented as mean ± SD (*n* = 3). (**E**) Transmission electron microscopy (TEM) image of sP-Lips@DR, scale bar: 200 nm.

Like DOX, R848 is a weakly basic drug, which means it may be encapsulated in the internal aqueous phase of liposomes via the remote loading method [35]. Therefore, we attempted to co-load DOX and R848 into sP-Lips. As illustrated in Figure 2A and Table S4, the EE of DOX in sP-Lips@DR (simultaneous loading) was only 67.37%, which was far lower than that of sP-Lips@DOX (94.35%). In addition, compared with sP-Lips@R (EE of R848: 23.18%), the EE of R848 in sP-Lips@DR (simultaneous loading) was reduced by nearly 2.63-fold (8.83%). These results suggest that simultaneous loading of DOX and R848 using an ammonium sulfate gradient reduced the EE of both drugs. Thus, we next attempted a sequential loading process, in which DOX and R848 were sequentially added in a time interval of 40 min. As shown in Figure 2A, the EE of DOX and R848 was restored to levels comparable to those achieved during single-drug loading, reaching 92.51% and 26.71%, respectively. These results indicate that the sequential loading method facilitates the efficient co-encapsulation of DOX and R848 in sP-Lips.

The increased co-encapsulation efficiency of DOX and R848 through sequential loading was attributed to their different loading mechanisms. During remote loading, DOX is trapped within the liposomes by forming (DOX-NH_3_)_2_SO_4_ precipitates [37]. Notably, as previously reported, the formation of the (DOX-NH_3_)_2_SO_4_ precipitate requires an ammonium sulfate concentration ≥150 nM [43]. For the simultaneous loading method, coexisting R848 may suppress the DOX precipitation by consuming ammonium sulfate. Therefore, sequential loading led to a higher DOX encapsulation efficiency than simultaneous loading. For R848, the previous study reported that FeSO_4_ induced the highest EE in liposomes among 5 trapping agents, including FeSO_4_, copper sulfate, ammonium sulfate, ammonium citrate, and aluminum sulfate [44]. This study found that ammonium sulfate had a poor R848-trapping effect. During simultaneous loading, a large amount of concomitant DOX can compete with R848 for binding to ammonium sulfate, thereby decreasing R848 EE.

The prepared sP-Lips@DR (sequential loading) were further characterized. As shown in Figure 2B–D and Table S5, the particles were well dispersed, with a mean diameter of 152.2 nm, a PDI value of 0.231, and a surface charge of −7.53 mV. Moreover, TEM images revealed that sP-Lips@DR were spherical and well-dispersed (Figure 2E).

### 3.3. Drug Release Behavior of sP-Lips@DR In Vitro

sPLA_2_ is overexpressed in HCC, with more than 5-fold higher levels than those in normal tissues [31]. Subsequently, the in vitro drug release behavior of sP-Lips@DR was evaluated in 10% FBS and sPLA_2_-containing PBS (pH 6.7) to mimic blood circulation and the sPLA_2_-rich, mildly acidic TME, respectively.

**Figure 3:**
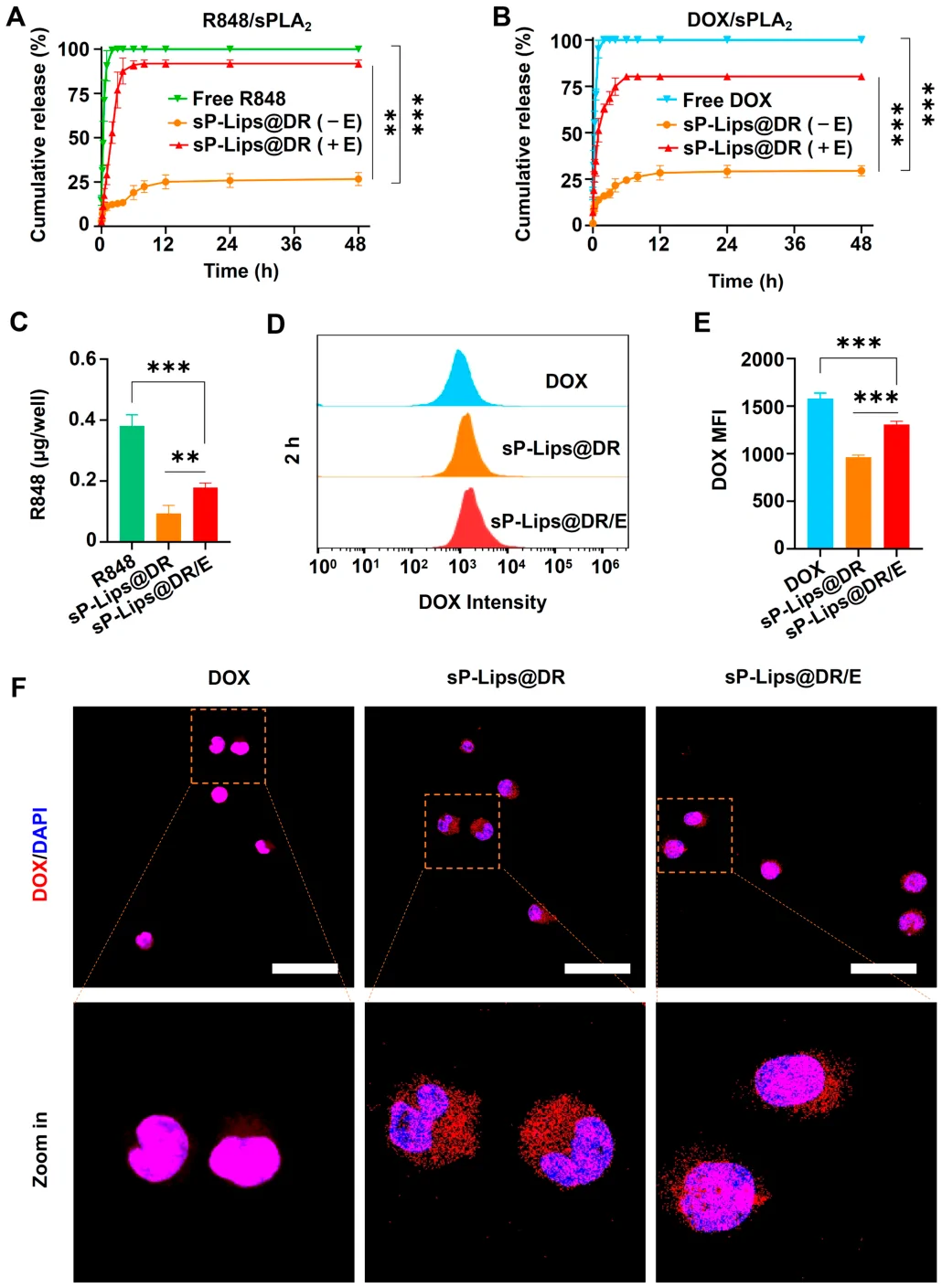
Drug release and sPLA_2_-responsive cellular uptake of sP-Lips@DR. (**A**,**B**) Cumulative release of (**A**) R848 or (**B**) DOX from sP-Lips@DR in different release media (*n* = 3). (**C**) Cellular uptake of R848 after 2 h incubation with free R848, sP-Lips@DR, or sP-Lips@DR in the presence of sPLA_2_ (sP-Lips@DR/E) (*n* = 3). (**D**) Flow cytometry histogram showing DOX cellular uptake in each group (free DOX, sP-Lips@DR, or sP-Lips@DR/E) after 2 h of incubation. (**E**) Median fluorescence intensity (MFI) of DOX analyzed from Panel (**D**) (*n* = 3). (**F**) Confocal imaging of H22 cells after 2 h of incubation with free DOX, sP-Lips@DR, or sP-Lips@DR/E (DOX: 0.5 μg/mL). The nuclei were stained with DAPI. Scale bar: 50 μm. Data were represented as mean ± SD. Statistical significance was calculated via a nonparametric two-tailed analysis of variance, where ** *p* < 0.01 and *** *p* < 0.001.

As shown in Figure 3A, the cumulative release of free R848 reached 99.44% within 2 h, indicating that the semipermeable membrane allowed free R848 to diffuse freely. However, the sP-Lips@DR (−) group exhibited a slow release profile in 10% FBS, with only 26.76% of R848 released after 48 h. These results suggest that the liposome is stable in blood circulation, thereby preventing premature R848 leakage. In contrast, R848 release was markedly accelerated in the sP-Lips@DR (+) group, with cumulative release reaching 87.63% at 4 h, indicating an accelerated drug release profile of sP-Lips@DR under sPLA_2_-rich, mildly acidic conditions. Similarly, the cumulative DOX release reached 74.90% at 4 h, which was 2.54-fold higher than that observed in 10% FBS at 48 h (Figure 3B). These results suggest that the sPLA_2_-containing, mildly acidic environment set to simulate the TME also promoted the release of DOX from sP-Lips@DR.

### 3.4. sPLA_2_-Promoted Cellular Uptake of sP-Lips@DR In Vitro

In addition, DOX and R848 need to be internalized by cells to exert their efficacy. Next, we evaluated whether enzyme-triggered drug release from sP-Lips@DR contributes to drug uptake by H22 and RAW 264.7 cells.

As shown in Figure 3C, RAW 264.7 cells exposed to free R848 internalized a markedly greater amount of R848 (0.38 μg) than those treated with sP-Lips@DR (0.09 μg), likely due to the ability of R848 to readily traverse the cell membrane. Notably, in the presence of sPLA_2_, R848 uptake in the sP-Lips@DR group increased markedly, reaching approximately 1.90-fold that observed in the sP-Lips@DR group without sPLA_2_. This result indicates that sPLA_2_-triggered R848 release from sP-Lips@DR promoted the intracellular accumulation of R848. Similarly, the median fluorescence intensity (MFI) of DOX in sP-Lips@DR (+)-treated H22 cells was 1.36-fold that of the sP-Lips@DR group, confirming the enzyme-triggered cellular uptake (Figure 3D,E).

Moreover, the internalization and distribution of sP-Lips@DR in H22 cells were visualized through confocal imaging. As shown in Figure 3F, in the free DOX group, the red fluorescence signal was predominantly localized in the nucleus, suggesting that DOX penetrated the H22 cell membrane for nuclear accumulation. In contrast, in the sP-Lips@DR group, the red fluorescence signal was mainly distributed in the cytoplasm, confirming that sP-Lips@DR entered H22 cells via endocytosis. Notably, in the presence of sPLA_2_, strong red fluorescence was observed in the nuclei of H22 cells, indicating that enzyme-responsive DOX release promoted its nuclear accumulation.

Further, to clarify the site of DOX release, we incubated DiD-labeled sP-Lips@DR with H22 cells with or without sPLA_2_, followed by confocal imaging of the cells and extracellular medium. In the sP-Lips@DR group, strong DiD (green)–DOX (red) co-localization (yellow) was observed and primarily distributed in the cytoplasm, indicating the internalization of sP-Lips@DR as a whole (Figure S4A). In contrast, in the presence of sPLA_2_, co-localization substantially decreased. DiD fluorescence (green) appeared surrounding the cells, and DOX fluorescence (red) accumulated in the nuclei, suggesting that most DOX was released from the liposomes inside the cells. Consistently, in the confocal images of the extracellular medium, the sP-Lips@DR group showed a higher DiD–DOX co-localization level (co-localization coefficient: 0.68) than sP-Lips@DR/E (co-localization coefficient: 0.14), confirming sPLA_2_-triggered DOX release from the liposomes (Figure S4B). Overall, these results suggest that sPLA_2_ triggers DOX release from sP-Lips@DR into the extracellular space for subsequent cell uptake.

### 3.5. sPLA_2_-Responsive Modulation of M2 Macrophages by sP-Lips@DR In Vitro

TAMs are abundant in HCC and predominantly exhibit an immunosuppressive M2 phenotype [13]. Since M2 TAMs inhibit DC maturation and T cell activation, reprogramming M2 TAMs into the antitumor M1 phenotype may help stimulate a robust ISV effect.

**Figure 4:**
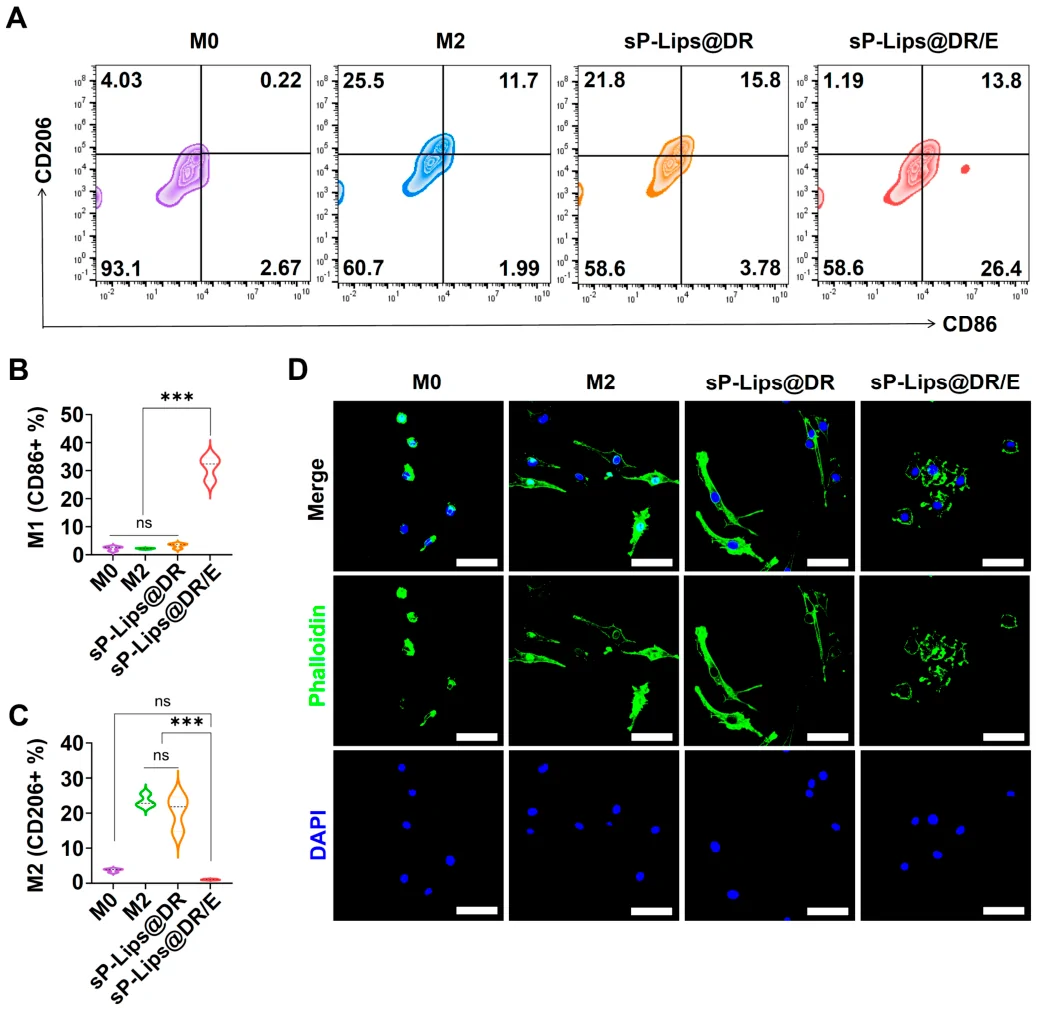
sP-Lips@DR modulated M2-like macrophages toward an M1-like phenotype in an sPLA_2_-dependent manner. (**A**) Representative flow cytometry contour plots of M1 and M2 macrophages after 48 h of incubation with sP-Lips@DR or sP-Lips@DR plus sPLA_2_ (sP-Lips@DR/E). M0 macrophages served as the non-polarized control, while PBS-treated M2 macrophages were used as the negative control. (**B**,**C**) The frequency of (**B**) M1 macrophages and (**C**) M2 macrophages in each group. (**D**) Confocal imaging of sP-Lips@DR-induced phenotypic modulation of M2-like macrophages toward an M1-like phenotype in the presence or absence of sPLA_2_. F-actin was stained with FITC-conjugated phalloidin to visualize the cellular morphology. Scale bar: 50 μm. Data were represented as mean ± SD (*n* = 3). Statistical significance was calculated via a nonparametric two-tailed analysis of variance, where *** *p* < 0.001 and ns, no significant difference.

To evaluate the modulatory effect of sP-Lips@DR on the phenotype of M2-like macrophages, M2-like macrophages were incubated with PBS, sP-Lips@DR, or sP-Lips@DR + sPLA_2_ for 48 h. As shown in Figure 4A–C, in the absence of sPLA_2_, sP-Lips@DR exhibited extremely low repolarization efficiency, with 20.40% M2 macrophages (CD206^+^CD86^−^) and only 3.23% M1 macrophages (CD206^−^CD86^+^), which was not significantly different from the PBS group. In contrast, treatment with sP-Lips@DR in the presence of sPLA_2_ markedly increased the percentage of M1 macrophages (31.13%), while decreasing the percentage of M2 macrophages (1.03%). These results indicate that sP-Lips@DR exhibits an sPLA_2_-triggered modulatory effect on M2-like macrophages, promoting a phenotypic shift toward an M1-like state.

In addition to characteristic markers, M2-like macrophages typically show an elongated, spindle-shaped morphology with slender protrusions, whereas M1-like macrophages tend to adopt a rounded, flattened, and polygonal morphology [20]. Phalloidin staining was performed to outline cell contours. As shown in Figure 4D, M0 RAW 264.7 cells exhibited a smooth, spherical morphology, whereas the cells pre-stimulated with IL-4, IL-10, and M-CSF displayed an elongated M2-like morphology. In contrast to the sP-Lips@DR group, the addition of sPLA_2_ induced a flattened and polygonal morphology characteristic of M1-like macrophages. In combination with the marker analysis, these morphological changes further supported the sPLA_2_-responsive modulation of M2-like RAW 264.7 macrophages toward an M1-like phenotype by sP-Lips@DR.

### 3.6. sPLA_2_-Responsive Cytotoxicity of sP-Lips@DR In Vitro

**Figure 5:**
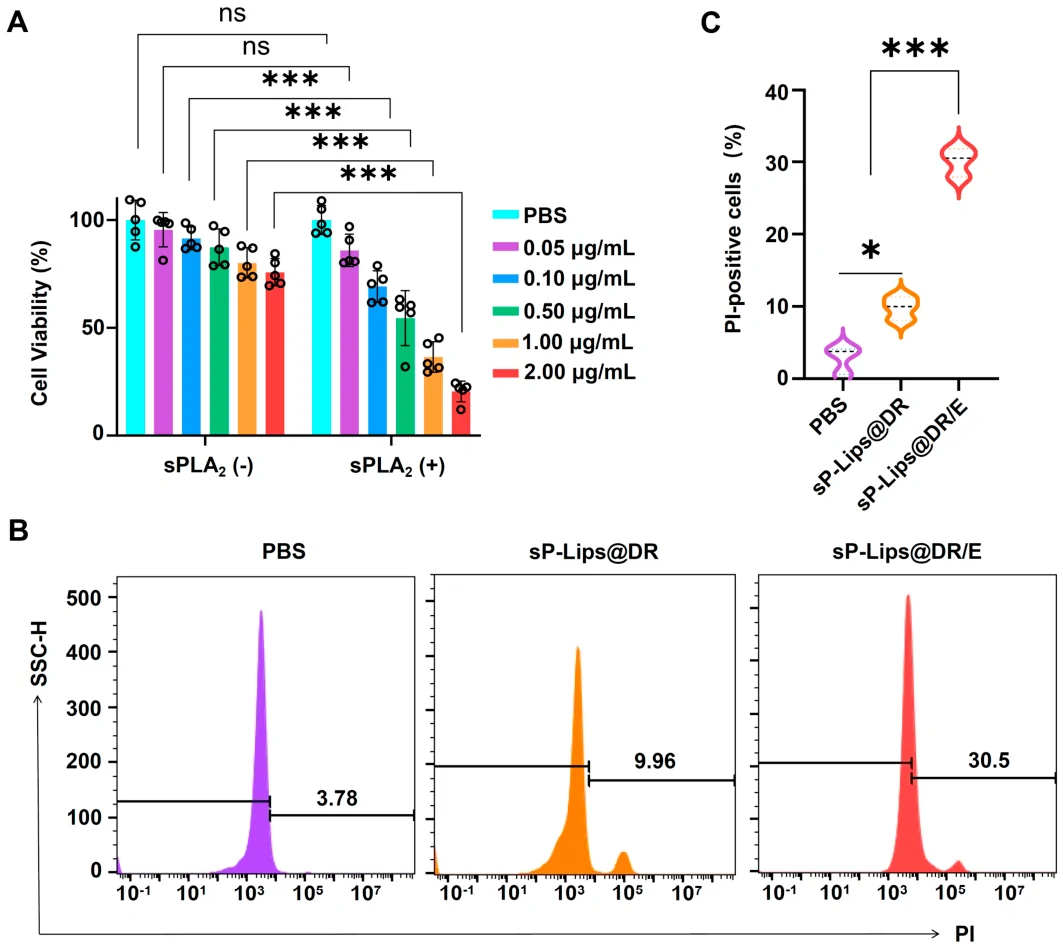
sPLA_2_-responsive cytotoxicity of sP-Lips@DR in H22 cells. (**A**) Cell viability of H22 cells after 24 h of treatment with sP-Lips@DR at various DOX concentrations in the presence or absence of sPLA_2_ (*n* = 5). (**B**) Representative flow cytometry histogram of H22 cells stained with PI after treatment with PBS, sP-Lips@DR, or sP-Lips@DR/E (DOX: 1 μg/mL). E: sPLA_2_. (**C**) Quantitative analysis of the percentage of PI-positive dead H22 cells from Panel (**B**) (*n* = 3). Data were represented as mean ± SD. Statistical significance was analyzed via a nonparametric two-tailed analysis of variance, where * *p* < 0.05, *** *p* < 0.001, and ns, no significant difference.

DOX possesses intrinsic cytotoxicity towards cancer cells [45]. A CCK-8 assay was performed to evaluate the sPLA_2_-responsive cytotoxicity of sP-Lips@DR against H22 cells. As shown in Figure 5A, in the absence of sPLA_2_, sP-Lips@DR exhibited low cytotoxicity toward H22 cells, with cell viability of over 85% at a DOX concentration of 0.5 μg/mL. In contrast, in the presence of sPLA_2_, sP-Lips@DR exhibited an increase in cytotoxicity at each concentration, particularly reducing H22 cell viability to 54.49% at a DOX concentration of 0.5 μg/mL.

To evaluate the potential cytotoxicity of sP-Lips@DR toward macrophages, we performed a CCK-8 assay in RAW 264.7 cells at various DOX concentrations in the presence or absence of sPLA_2_. As DOX concentrations increased, cell viability decreased, whether or not sPLA_2_ was present, but was not significantly different from the PBS control, indicating negligible cytotoxicity to RAW 264.7 cells. In particular, cell viability remained 95.87% and 100.83% at a DOX concentration of 0.5 μg/mL in the presence and absence of sPLA_2_, respectively. The concentration of 0.5 μg/mL was used in the cellular experiments, thereby precluding potential cytotoxicity of sP-Lips@DR toward RAW 264.7 cells. In addition, the cell viability of the sPLA_2_ (92.73%) and sPLA_2_ + sP-Lips (91.39%) groups was comparable to the PBS control (100%), indicating little cytotoxic effect on RAW 264.7 cells.

DOX is commonly known as an ICD-inducing agent. Thus, DOX-induced tumor cell apoptosis is often accompanied by the release of damage-associated molecular patterns (DAMPs) and immune activation [46,47]. Propidium iodide (PI) staining is typically used to distinguish live cells from late-stage apoptotic or necrotic cells [48]. Herein, PI staining was performed to assess whether sP-Lips@DR exhibited sPLA_2_-dependent cell-killing activity. The percentage of PI-positive dead H22 cells in the sP-Lips@DR group (9.79%) was significantly higher than in the PBS control (2.80%) (Figure 5B,C). In contrast, the addition of sPLA_2_ further improved the percentage of dead cells to 30.07%, with a 3.07-fold increase. These results indicated that sP-Lips@DR exhibited an sPLA_2_-responsive killing effect on tumor cells.

### 3.7. In Vitro Disruption Effects of DOX/R848 on the M2 TAM-H22 Cell Crosstalk

The previous study has reported a crosstalk between M2 TAMs and liver cancer cells, in which cancer cells reinforce the M2 phenotype of TAMs [13], thereby impairing ISV efficacy. Here, we evaluated whether DOX combined with R848 could disrupt the crosstalk by exerting cytotoxic effects on cancer cells and promoting a phenotypic shift in M2-like TAMs toward an M1-like state, respectively.

**Figure 6:**
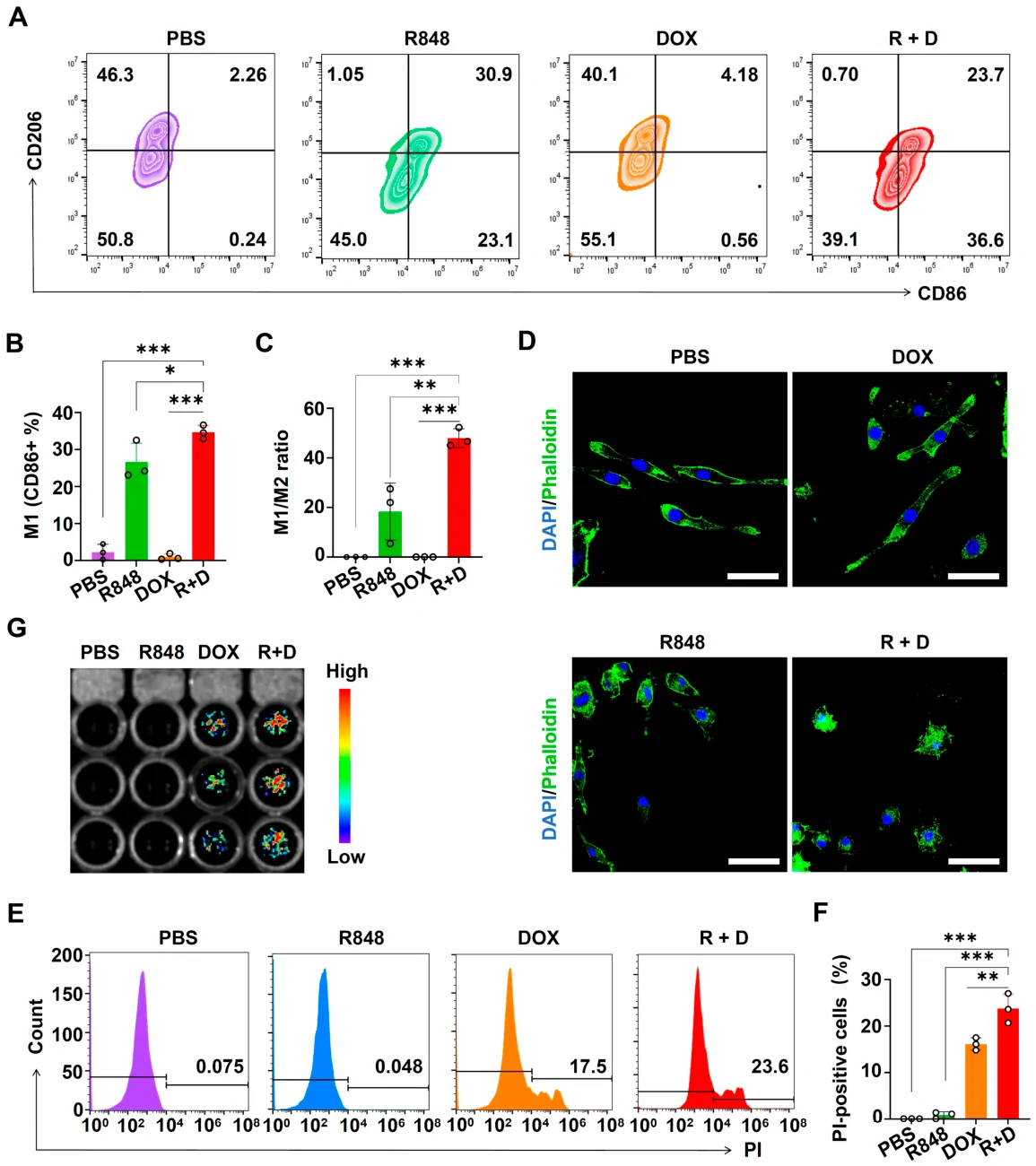
Evaluation of the disruption effect of R848/DOX on the M2 TAM-H22 cell crosstalk. (**A**) Representative flow cytometry contour plots of M1 and M2 macrophages after treatment with the indicated formulations. R + D: R848 plus DOX. (**B**) The frequency of M1 macrophages and the (**C**) M1/M2 ratio were analyzed from Panel (**A**). (**D**) Representative confocal images of the macrophages after different treatments. F-actin was stained with FITC-conjugated phalloidin to visualize the cellular morphology. Scale bar: 50 μm. (**E**) Representative flow cytometry histogram of H22 cells stained with PI after various treatments. (**F**) Quantitative analysis of the percentage of PI-positive dead H22 cells in Panel (**E**). (**G**) H22 cells were stained with PI, and the fluorescence intensity of PI was detected using an in vivo imaging system. Data were represented as mean ± SD (*n* = 3). Statistical significance was calculated via a nonparametric two-tailed analysis of variance. * *p* < 0.05, ** *p* < 0.01, and *** *p* < 0.001.

As shown in Figure 6A–C, the percentage of M1 macrophages in the DOX group (0.94%) showed no significant difference from the PBS group (2.29%), indicating that DOX alone failed to induce a phenotypic shift in macrophages toward an M1-like state. In contrast, treatment with R848 led to an M1 proportion of 26.60%, which was 11.62-fold higher than that in the PBS group, demonstrating its potent macrophage-modulatory activity. Notably, the addition of DOX further elevated the M1 macrophage proportion to 34.67%, yielding a 2.62-fold greater M1/M2 ratio than that in the R848 group. Consistently, compared with the R848 group, the addition of DOX further increased the number of rounded and flattened M1-like cells (Figure 6D). These results indicate that DOX disrupted the H22 cell-mediated reinforcement of the M2-type TAM phenotype by killing H22 cells.

The percentage of PI-positive dead H22 cells in the R848 group (0.85%) was comparable to that in the PBS group (0.069%), showing its negligible effect on H22 cells’ viability (Figure 6E,F). DOX increased the percentage of dead cells to 16.13% due to its intrinsic cytotoxicity. Interestingly, the highest percentage of PI-positive dead cells was observed in the DOX/R848 combination group (23.77%). Consistently, the combination group displayed the strongest PI fluorescence signal, indicating the highest cell death (Figure 6G). These results suggest that R848-mediated M2-to-M1 repolarization weakened the pro-survival effect of M2 TAMs on H22 cells. Therefore, it could be deduced that DOX combined with R848 could effectively disrupt the M2 TAMs-H22 cell crosstalk.

### 3.8. Evaluation of sPLA_2_-Responsive Disruption Effect of sP-Lips@DR on the Crosstalk

**Figure 7:**
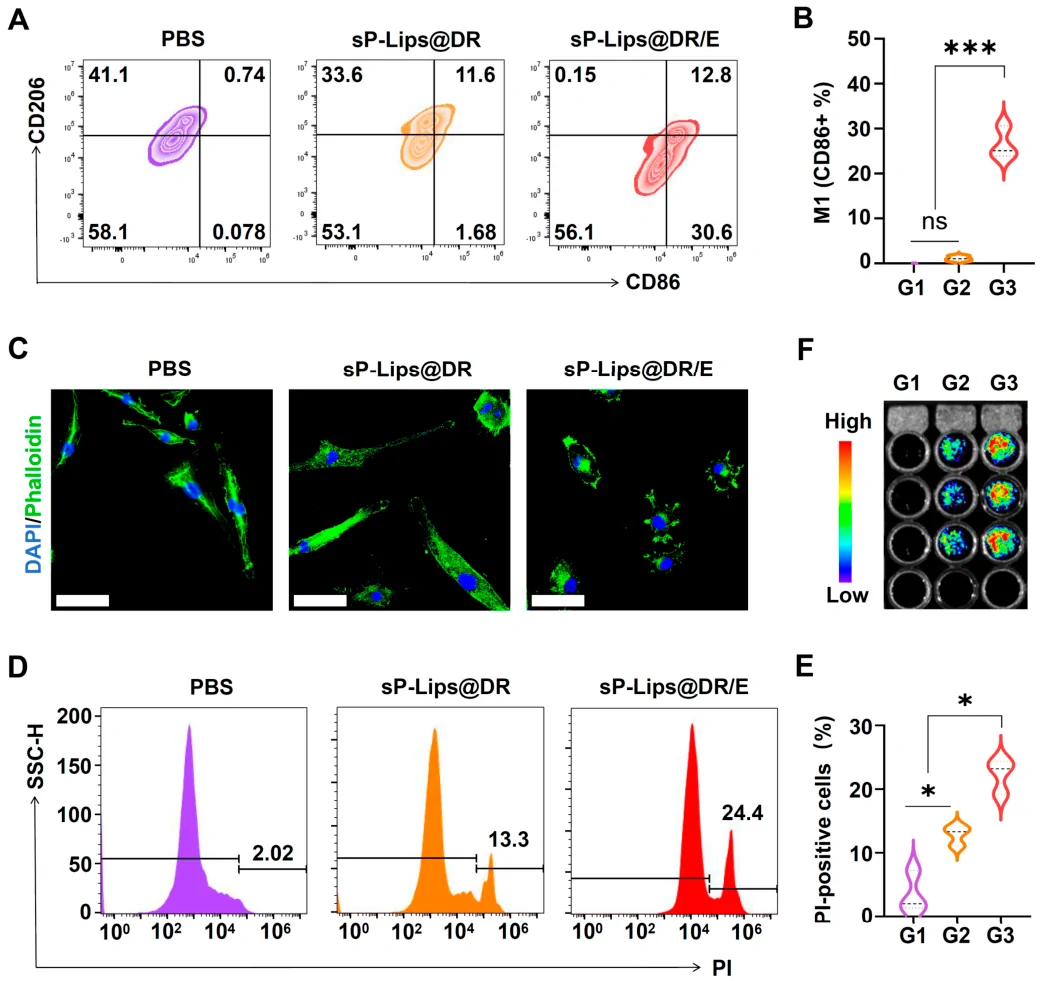
Evaluation of sPLA_2_-dependent modulation effects of sP-Lips@DR on the co-cultured TAMs and H22 cells. (**A**) Representative flow cytometry contour plots of M1 and M2 macrophages after 48 h incubation with PBS, sP-Lips@DR, or sP-Lips@DR + sPLA_2_ (sP-Lips@DR/E) in a Transwell system. (**B**) The frequency of M1 macrophages analyzed from Panel (**A**). (**C**) Representative confocal images of the macrophages after cytokine-polarized RAW 264.7 macrophages were subjected to various treatments. F-actin was stained with FITC-conjugated phalloidin to visualize cell morphology. Scale bar: 50 μm. (**D**) Representative flow cytometry histogram of H22 cells stained with PI after different treatments and (**E**) quantitative analysis of the percentage of PI-positive dead H22 cells. (**F**) H22 cells were treated with various formulations and stained with PI. The fluorescence intensity of H22 cells was then detected by an in vivo imaging system. G1: PBS, G2: sP-Lips@DR, G3: sP-Lips@DR/E. Data were represented as mean ± SD (*n* = 3). Statistical significance was calculated via a nonparametric two-tailed analysis of variance. * *p* < 0.05, *** *p* < 0.001, and ns, no significant difference.

Given that the DOX/R848 combination could disrupt the intercellular crosstalk, we next evaluated whether sP-Lips@DR exerted an sPLA_2_-responsive modulation effect on both cells’ interaction [49]. In the sP-Lips@DR group, the proportion of M1 macrophages was only 1.08%, which was slightly higher than that in the PBS group (0.053%) (Figure 7A,B and Figure S3). This result indicated that sP-Lips@DR alone is inefficient in inducing a phenotypic shift in M2-like macrophages toward an M1-like state. In contrast, in the sP-Lips@DR + sPLA_2_ group, the proportion of M1 macrophages was 26.53%, representing a 24.57-fold increase relative to the sP-Lips@DR group, suggesting that the incorporation of sPLA_2_ markedly enhanced the modulatory effect of sP-Lips@DR on the TAM phenotype. Consistently, confocal imaging showed that the macrophages in the sP-Lips@DR + sPLA_2_ group presented a rounded and polygonal morphology, further confirming the sPLA_2_-responsive phenotypic shift toward an M1-like state (Figure 7C).

In addition, the percentage of PI-positive H22 cells in the sP-Lips@DR + sPLA_2_ group was 1.74-fold higher than that in the sP-Lips@DR group, indicating the sPLA_2_-dependent cell-killing activity of sP-Lips@DR (Figure 7D,E). Moreover, the sP-Lips@DR + sPLA_2_ group exhibited a much stronger PI fluorescence signal than other groups, indicating a larger proportion of H22 cells undergoing death (Figure 7F). Taken together, these results demonstrate that sP-Lips@DR exerts sPLA_2_-dependent modulation effects on the co-cultured M2-like macrophages and H22 cells.

## 4. Conclusions

In this study, sPLA_2_-responsive dual-drug-loaded liposomes, sP-Lips@DR, were successfully fabricated. The incorporation of CHOL significantly improved the encapsulation efficiency of DOX, while a sequential loading process enabled efficient co-encapsulation of DOX and R848. The resulting sP-Lips@DR exhibited a uniform particle size and good dispersion. Importantly, after responding to sPLA_2_, sP-Lips@DR rapidly released DOX and R848 and promoted their uptake by target cells. Functionally, sP-Lips@DR showed sPLA_2_-dependent modulation of M2 macrophages toward an M1-like phenotype, as well as cytotoxicity against H22 cells. Moreover, we verified that sP-Lips@DR exhibited sPLA_2_-responsive disruption effects on intercellular crosstalk. Overall, this study provides a robust experimental basis for further evaluation of the potential of sP-Lips@DR as an in vivo ISV nanoplatform for HCC.

## Figures and Tables

**Table 1:** Lipid formulations of the various liposomes.

Liposomes	DPPC (mol%)	CHOL (mol%)	DSPE-PEG_2000_ (mol%)
sP-Lips (F1)	95	/	5
sP-Lips (F2)	75	20	5

## Data Availability

The data are available from the corresponding author upon reasonable request.

## Supplementary Materials

The following supporting information can be downloaded at https://www.mdpi.com/article/10.3390/pharmaceutics18080964/s1: Figure S1: Screening of ultracentrifugation force for sP-Lips@DiD (F1); Figure S2: Screening of ultracentrifugation force for sP-Lips@DiO (F2); Figure S3: The frequency of M2 TAMs in PBS, sP-Lips@DR, and sP-Lips@DR/E groups; Figure S4: sPLA_2_-responsive cellular uptake of sP-Lips@DR; Figure S5: Cell viability of RAW 264.7 cells after 24 h of incubation with sP-Lips@DR at various DOX concentrations in the presence or absence of sPLA_2_ (*n* = 5); Table S1: Particle size and zeta potential of sP-Lips (F1) and sP-Lips (F2); Table S2: Lipid formulations of sP-Lips@DiD (F1) and sP-Lips@DiO (F2); Table S3: Recovery efficiency of sP-Lips@DiD (F1) and sP-Lips@DiO (F2); Table S4: DLC and EE of DOX in sP-Lips@DOX (F1) and sP-Lips@DOX (F2); Table S5: Particle size and zeta potential of drug-loading liposomes.
