# Supplementary material for: Dual Drug-Loaded Enzyme-Responsive Liposomes Exert Specific Modulation on Liver Cancer Cells and Tumor-Associated Macrophages In Vitro
Source: Pharmaceutics. 2026 Aug 5;18(8):964. doi: 10.3390/pharmaceutics18080964 (PMC13516798; doi:10.3390/pharmaceutics18080964)
Supplement: Supplementary file 1 [file pharmaceutics-18-00964-s001.zip › pharmaceutics-4452714-supplementary.pdf]

# Supporting Information

# Dual drug-loaded enzyme-responsive liposomes exert specific modulation on liver cancer cells and tumor-associated macrophages in vitro

Shudong Zhang [1,2,†], Jun Yan [1,2,†], Shiyu Zhu [1,2], Xinchen Liu [1,2], Lele Wang [1,2], Yuhang Liu [1,2] and Yan Wei [1,2,*]

[1] Institute of Translational Medicine, Shanghai University, Shanghai 200444, China

[2] MedEng-X Institutes, Shanghai University, Shanghai 200444, China

† These authors contributed equally to this work.

* Corresponding author: ywei@shu.edu.cn

# 1. Materials and Methods

*1.1. Materials*

1,2-dipalmitoyl-sn-glycero-3-phosphocholine (DPPC) and Cholesterol (CHOL) were ordered from A.V.T. Pharmaceutical Co., Ltd. (Shanghai, China). 1,2-distearoyl-sn-glycero-3-phosphoethanolamine-N-[methoxy (polyethylene glycol)-$_{2000}$] (DSPE-$PEG_{2000}$) was purchased from Bide Pharmatech Co., Ltd. (Shanghai, China). 3,3′-dioctadecyloxacarbocyanine perchlorate (DiO), 1,1-dioctadecyl-3,3,3′,3′-tetramethy lindodicarbocyanine 4-chlorobenzenesulfonate salt (DiD), 2-(4-Aminophenyl)-6-indolecarbamidine dihydrochloride (DAPI), a CCK8 kit, and an ATP assay kit were obtained from Beyotime Biotechnology Co., Ltd. (Shanghai, China). Sigma APC anti-mouse CD206 rabbit recombinant antibody and PE anti-mouse CD86 antibody were obtained from Wuhan SANYING Biotechnology, Inc. (Wuhan, China). Secretory phospholipase A2 ($sPLA_2$) from porcine pancreas was obtained from Sigma-Aldrich Trading Co., Ltd. (Shanghai, China). Propidium iodide staining solution (PI) and TRITC Phalloidin were purchased from Beijing Solarbio Science & Technology Co., Ltd. (Beijing, China). Doxorubicin (DOX) and Resiquimod (R848) were purchased from AbMole Bioscience Co., Ltd. (Shanghai, China). All other chemicals were purchased from Sinopharm Chemical Reagent Co., Ltd. (Shanghai, China) unless otherwise stated.

*1.2. Cell Lines*

The mouse macrophage cell line RAW 264.7 and mouse-derived hepatocellular carcinoma cell line H22 were provided by Zhong Qiao Xin Zhou Biological Technology Co., Ltd. (Shanghai, China). The cells were cultured in RPMI-1640 medium supplemented with 10% FBS, penicillin (100 U/mL), and streptomycin (100 μg/mL)

and maintained at 37 °C in a 95% humidified atmosphere with 5% $CO_2$.

*1.3. Optimization of Ultracentrifugation Speed for Liposomes*

To separate drug-loaded liposomes from unencapsulated drugs, the ultracentrifugation speed was screened. Briefly, sP-Lips@DiD (F1) and sP-Lips@DiO (F2) were prepared using the thin-film hydration method by adding DiD or DiO to the lipid components at a dye-to-lipid molar ratio of 1:100. Following hydration, the liposome suspension was sonicated to reduce the particle size. The liposome suspensions were ultracentrifuged at different centrifugal forces, including 150,000 × g and 200,000 × g, for 2 h. The liposomal pellets were resuspended in PBS, and the fluorescence intensities of DiD or DiO were measured using a microplate reader (Infinite® 200 PRO, Tecan, Männedorf, Switzerland).

*1.4. Evaluation of $sPLA_2$-responsive Cellular Uptake of sP-Lips@DR*

*1.4.1. Evaluation of Cellular Uptake Efficiency*

For cellular uptake of R848, RAW 264.7 cells ($1.0 \times 10^5$ cells per well) were seeded into 12-well plates and incubated overnight. R848, sP-Lips@DR, or sP-Lips@DR + $sPLA_2$ (sP-Lips@DR/E) was then added to each well and incubated for 2 h. Then, the cells were washed with PBS and collected by centrifugation at 1000 rpm for 5 min. The cell pellets were lysed with methanol/water (3:1, v/v), sonicated for 10 min, and then centrifuged at 10,000 rpm for 10 min. The fluorescence intensity of R848 in the supernatants was measured with a microplate reader (Infinite® 200 PRO, Tecan, Männedorf, Switzerland).

For cellular uptake of DOX, H22 cells ($1.0 \times 10^5$ cells per well) were incubated in 12-well plates. DOX, sP-Lips@DR, or sP-Lips@DR/E was then added to each well and incubated for 2 h. Cellular uptake of DOX in H22 cells was quantified by flow cytometry assay (Cytoflex LX, Beckman, Indianapolis, IN, USA), and the data were

analyzed using FlowJo software (v10.8.1).

*1.4.2. Cellular Uptake and Intracellular Localization*

After incubation as described above, H22 cells were washed with PBS, fixed in 4% paraformaldehyde, and stained with DAPI for 10 min. Intracellular DOX localization was then observed using confocal laser scanning microscopy (CLSM) (FV3000, Olympus, Tokyo, Japan).

To further investigate the site of DOX release, H22 cells were incubated with free DOX, DiD-labeled sP-Lips@DR, or DiD-labeled sP-Lips@DR + $sPLA_2$ (sP-Lips@DR/E) for 2 h. The extracellular medium was separated from the cells by centrifugation. The extracellular medium or cells were imaged using CLSM.

*1.5. $sPLA_2$-Responsive Modulation of M2-Like TAMs toward an M1-Like Phenotype by sP-Lips@DR*

RAW 264.7 cells were seeded into the lower chambers of a 12-well Transwell plate (0.4 μm) and incubated overnight. To induce M2 polarization, the cells were treated with IL-4 (0.5 μg/mL), IL-10 (0.25 μg/mL), and M-CSF (0.25 μg/mL) for 48 h. The polarized cells were subsequently treated with PBS, sP-Lips@DR, or sP-Lips@DR/E for 48 h. RAW 264.7 cells (M0) served as controls. Then, the cells were resuspended in PBS and incubated with anti-mouse CD16/32 antibodies (1 μg per $10^6$ cells) for 10 min at 4 °C to block Fc receptors. The cells were stained with APC anti-mouse CD206 and PE anti-mouse CD86, and the distribution of macrophage subsets (M1/M2) was analyzed by cytometry assay (Cytoflex LX, Beckman, Indianapolis, IN, USA).

In addition, F-actin was stained with FITC-conjugated phalloidin to visualize cellular morphology. The nuclei were stained with DAPI. The cells were imaged using CLSM (FV3000, Olympus, Tokyo, Japan).

*1.6. $sPLA_2$-Responsive Cytotoxicity of sP-Lips@DR against H22 cells*

*1.6.1. Evaluation of the Cytotoxicity of sP-Lips@DR against H22 Cells Using the CCK-8 Assay*

H22 cells were seeded in a 96-well plate at a density of 8 × $10^3$ cells/well and incubated overnight. The cells were treated with sP-Lip@DR at various DOX concentrations, with $sPLA_2$ ($sPLA_2$ (+)) or without $sPLA_2$ ($sPLA_2$ (-)). After 24 h of treatment, 10 μL of CCK-8 solution was added to each well, followed by incubation at 37 °C for 4 h. The absorbance at 450 nm was measured using a microplate reader (Infinite® 200 PRO, Tecan, Männedorf, Switzerland). Cell viability was analyzed using GraphPad Prism 8.4.3.

*1.6.2. Detection of PI-positive Dead Cells through Flow Cytometry*

H22 cells (1 × $10^5$ cells/well) were seeded into the lower chambers of a 12-well Transwell plate and incubated overnight. Then, PBS, sP-Lips@DR, or sP-Lips@DR/E (DOX concentration: 0.5 μg/mL) was added to the upper chamber and incubated for 24 h. Then, H22 cells were incubated with 10 μg/mL PI in PBS for 30 min. The cells were suspended in PBS, and fluorescence intensity was measured through flow cytometry (Cytoflex LX, Beckman, Indianapolis, IN, USA). The data were analyzed using FlowJo software (v10.8.1).

*1.7. Cytotoxicity of sP-Lips@DR toward RAW 264.7 cells*

Cytotoxicity of sP-Lips@DR toward RAW 264.7 cells was detected via a CCK-8 assay. Briefly, RAW 264.7 cells were seeded in 96-well plates at a density of 8 × $10^3$ cells/well and treated with sP-Lips@DR at various DOX concentrations in the presence or absence of $sPLA_2$. $sPLA_2$ alone ($sPLA_2$) and empty sP-Lips plus $sPLA_2$ ($sPLA_2$ + sP-Lips) groups served as controls. After 24 h of incubation, cell viability was measured using the CCK-8 assay by measuring the UV absorbance at 450 nm. Cell viability was analyzed using GraphPad Prism 8.4.3.

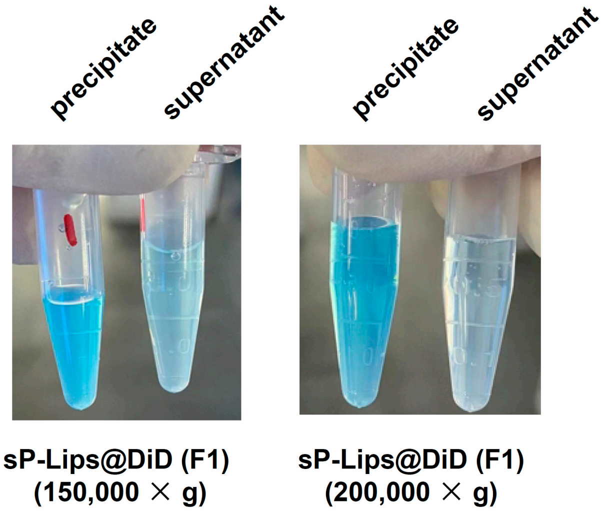


**Figure S1.** Screening of ultracentrifugation force for sP-Lips@DiD (F1). Photographs of the suspension and supernatant of sP-Lips@DiD (F1) after centrifugation at 150,000 × g and 200,000 × g, respectively.

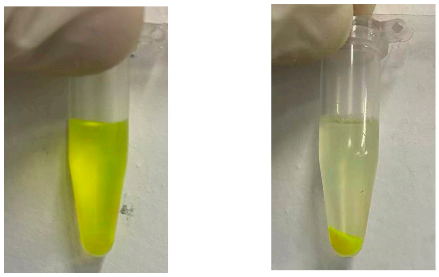

**sP-Lips@DiO (F2)**
**(150,000 × g)**

**sP-Lips@DiO (F2)**
**(200,000 × g)**

**Figure S2.** Screening of ultracentrifugation force for sP-Lips@DiO (F2). Photographs of sP-Lips@DiO (F2) after centrifugation at 150,000 × g and 200,000 × g, respectively.

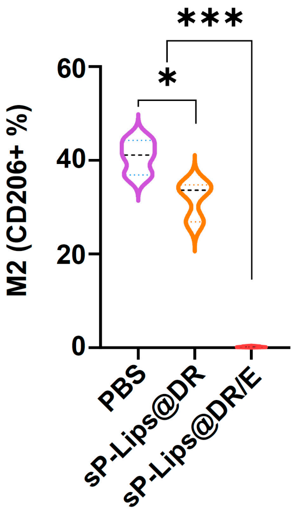


**Figure S3.** The frequency of M2 TAMs in PBS, sP-Lips@DR, and sP-Lips@DR/E groups. Data are presented as mean ± SD ($n$ = 3). Statistical significance was analyzed via a nonparametric two-tailed analysis of variance. *$p < 0.05$ and ***$p < 0.001$.

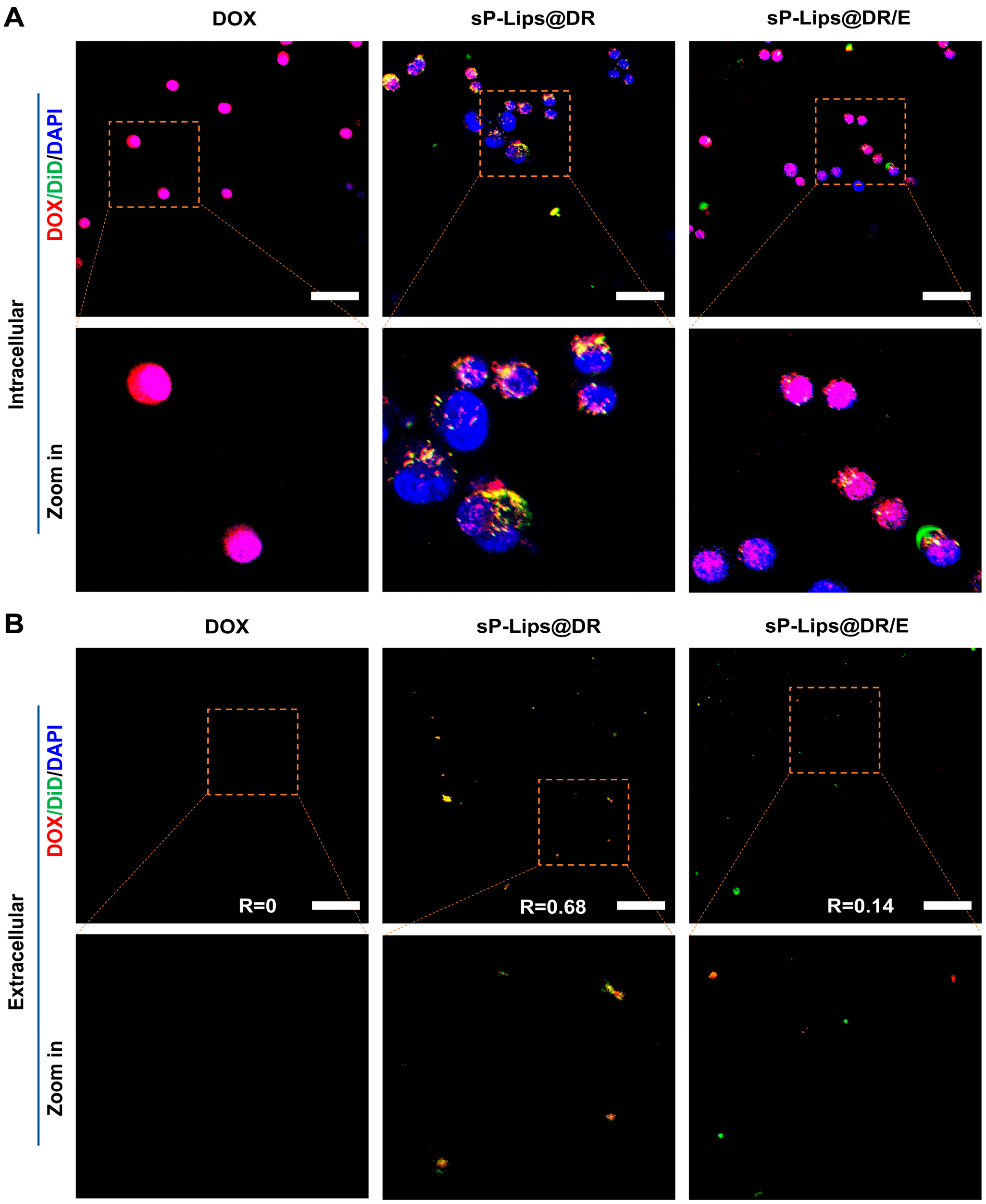


**Figure S4.** sPLA2-responsive cellular uptake of sP-Lips@DR. Confocal imaging of (A) H22 cells and (B) extracellular media after 2 h of incubation with free DOX, DiD-labeled sP-Lips@DR, or DiD-labeled sP-Lips@DR/E. The nuclei were stained with DAPI. Scale bars: 50 μm.

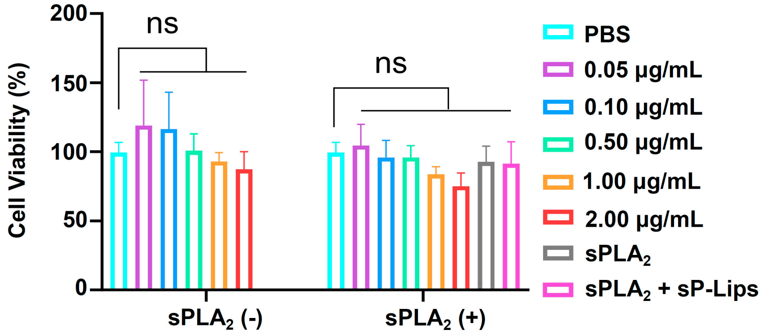


**Figure S5.** Cell viability of RAW 264.7 cells after 24 h of incubation with sP-Lips@DR at various DOX concentrations in the presence or absence of $sPLA_2$ ($n$ = 5). $sPLA_2$ alone and $sPLA_2$ combined with sP-Lips ($sPLA_2$ + sP-Lips) served as controls. Data are presented as mean ± SD. Statistical significance was analyzed via a nonparametric two-tailed analysis of variance. ns, no significant difference.

**Table S1.** Particle size and zeta potential of sP-Lips (F1) and sP-Lips (F2).

| **Liposomes** | Z-average (nm) | PDI | Zeta potential (mV) |
|---|---|---|---|
| sP-Lips (F1) | 118.5 ± 2.6 | 0.226 ± 0.016 | -6.87 ± 0.91 |
| sP-Lips (F2) | 146.4 ± 0.6 | 0.245 ± 0.009 | -7.23 ± 0.69 |

**Table S2.** Lipid formulations of sP-Lips@DiD (F1) and sP-Lips@DiO (F2).

| **Liposomes** | **DPPC (mol%)** | **CHOL (mol%)** | **DSPE-PEG$_{2000}$ (mol%)** | **DiD/DiO (mol%)** |
|---|---|---|---|---|
| sP-Lips@DiD (F1) | 95 | / | 5 | 1 |
| sP-Lips@DiO (F2) | 75 | 20 | 5 | 1 |

**Table S3.** Recovery efficiency of sP-Lips@DiD (F1) and sP-Lips@DiO (F2).

| **Liposomes** | **Centrifugal speed (g)** | **Recovery efficiency (%)** |
|---|---|---|
| sP-Lips@DiD (F1) | 150,000 | 65.54 ± 0.67 |
| sP-Lips@DiD (F1) | 200,000 | 94.02 ± 1.48 |
| sP-Lips@DiO (F2) | 150,000 | 71.93 ± 0.84 |
| sP-Lips@DiO (F2) | 200,000 | 96.74 ± 1.19 |

**Table S4.** DLC and EE of DOX in sP-Lips@DOX (F1) and sP-Lips@DOX (F2).

| Liposomes | DLC of DOX (%) | EE of DOX (%) |
|---|---|---|
| sP-Lips@DOX (F1) | 2.26 ± 0.14 | 15.08 ± 0.91 |
| sP-Lips@DOX (F2) | 10.38 ± 0.51 | 94.35 ± 4.61 |

**Table S5.** Particle size and zeta potential of drug-loading liposomes.

| **Liposomes** | Z-average (nm) | PDI | Zeta potential (mV) |
|---|---|---|---|
| sP-Lips@DOX | 147.2 ± 1.1 | 0.252 ± 0.006 | -6.27 ± 1.23 |
| sP-Lips@DR (Se-loading) | 152.2 ± 1.6 | 0.231 ± 0.011 | -7.53 ± 0.87 |
